# Rose-Bradford Kidneys

**Published:** 1907-08-24

**Authors:** 


### Illustrative Cases.

### ROSE-BRADFORD KIDNEYS.

There are two chief forms of chronic Bright's disease, namely, granular kidney and chronic tubal nephritis. The latter sometimes arises definitely out of an original acute nephritis, which was diagnosed during the acute stage, but which failed to clear up in spite of treatment; it may also arise insidiously, however, without there having been any symptoms of a preceding acute nephritis at all; the kidney found post-mortem is a small pale one, microscopical examination of which shows that there is both epithelial degeneration and chronic interstitial fibrosis. When a small pale kidney of this kind develops insidiously it is sometimes called a Rose-Bradford kidney, to distinguish it from a precisely similar kidney that is secondary to a known acute nephritis. We have elsewhere brought forward evidence in favour of the view that Rose-Bradford kidneys are really secondary to a preceding acute inflammation, which attracted no notice because there was no œdema at the time; but be this as it may, there is no doubt that there is, clinically, a variety of chronic tubal nephritis which arises quite insidiously, and it is to the small pale kidney found at the autopsy in such a case that we attach the name "Rose-Bradford."

How long the mischief has been present in these cases before symptoms develop it is impossible to say. Judging from the amount of fibrosis there is in microscopical sections of the organs, the latter must have been diseased for a long time, months certainly, and probably years.

The symptoms that arise from Rose-Bradford kidneys are in the main similar to those to which granular kidneys give rise, and according to the kind of symptoms that first appear we can distinguish the following clinical groups:—

1. Those cases commencing with failure of the eyesight from albuminuric retinitis.
2. Those with dyspnœa and heart failure.
3. Those with a severe hæmorrhage or apoplexy.
4. Those with symptoms of uræmia—chronic or acute.
5. Those in which an acute exacerbation of the nephritis occurs, so that unless great care is exercised the diagnosis will be primary acute nephritis instead of an acute exacerbation of a long existent latent nephritis.

The commonest of all the above groups is probably the last, though it is not of these that we propose to give examples to-day; we would, however, emphasise the fact that many a case in which the sudden onset of general œdema associated with albuminuria, hæmaturia, and casts seems to suggest an acute inflammation in kidneys hitherto healthy, is really a much graver condition—acute nephritis upon the top of chronic.

The next commonest group is the fourth, in which the first symptoms are those of uræmia developing in a person who hitherto had not only regarded himself as healthy, but who had been so regarded by all his friends. It is of these cases that we propose to give examples. It will be noticed that *the patients are comparatively young.* That the prognosis is bad, much worse than it is when granular kidney gives rise to its first symptoms. That as a rule there is much albumen in an abundant urine of low specific gravity; the third case we give is an exception to this, for the urine contained no albumen at all. That there is little or no œdema; that the patients are pale; and that the left ventricle of the heart is hypertrophied.

Case 1.—A girl, aged 13, had had measles years ago, and had once suffered from tape-worm, but had had no other illnesses, and had been strong and well in herself, though pale, until seven weeks before her death. She had not had scarlet fever as far as she knew, and she had never had any œdema, nor anything suggestive of an acute nephritis. Seven weeks before she died she began to have attacks of dyspnœa at intervals, but she did not see a doctor. The attacks were attributed to "asthma," but in view of the ultimate result there can be little doubt that they were not true asthma, but uræmic asthma. Three days before the end she had one of these dyspnœic attacks, which was so bad that medical attention was sought. There was a soft systolic bruit in the pulmonary area, apparently hæmic in origin, otherwise the heart seemed normal, except for a prolongation of the first sound at the impulse. The lungs and the alimentary and nervous systems were natural. The urine was abundant, pale, of low specific gravity, free from sugar, but loaded with albumen; under the microscope many hyaline and granular tube casts were seen, and a few renal cells, but no red corpuscles. There was no œdema at all. The eyes seemed normal on ophthalmoscopic examination.

The acute dyspnœa subsided into a deep sighing respiration not unlike air-hunger; the girl had been conscious at first, but she rapidly became comatose. She remained in this condition for two days, and then her heart seemed to dilate rapidly; just before the end vomiting set in, and there was blood in the vomit. There was no convulsion at any time, death ensuing quite quietly during the coma.

At the autopsy the right side of the heart was found much dilated and not hypertrophied, whilst the left ventricle was considerably hypertrophied and hardly dilated at all; the valves were healthy. There was no brain lesion. The only radically diseased organs were the kidneys. Each of the latter was very pale, almost white, with no adherence of the capsule, though the surface was slightly granular; the cortex was extremely thin, the pyramids were atrophied and contracted, and it was very difficult to define the junction between cortex and medulla; the pelves were not dilated, the ureters were healthy; the arterioles were not thickened, and there were no cysts; each kidney weighed just over ½ oz., and at the time of the autopsy the note was made: "each is about the size of an average oyster, *i.e.*, the oyster, not its shell." The two most remarkable things about them were (1) their pallor, (2) their small size. They were typical small white kidneys.

The report upon the microscopical characters of a section from one of the kidneys was as follows: "The organ is so affected by the diseased process that it is with difficulty recognisable as renal. There is a very great excess of interstitial fibrous tissue, much of which has undergone hyaline degeneration. The vessels are not particularly thickened. Only here and there can the outlines of a glomerulus be discerned; the tubules have largely disappeared; those which can still be distinguished are shrunken and constricted by the fibrous tissue."

It may be remarked that, although the condition has usually been termed chronic *tubal* nephritis, the main feature of the sections of these small white kidneys is always the great excess of the *interstitial* fibrous tissue with disappearance of many of the tubules.

Case 2.—A man, aged 32, had had measles as a child, but no scarlet fever; had served with the Army in India, where he had malaria badly; was of temperate habits, and denied all venereal trouble; had had "influenza" often during the last four years; but had not been "ill" until seven weeks before he first came under observation. He began to suffer from nausea and vomiting without apparent cause; this increased rather than diminished, headache and insomnia supervened, and latterly he had suffered from unaccountable attacks of severe dyspnœa at night, associated with polyuria and intense thirst. These were the symptoms of which he now complained. He was a big man, but very thin and anæmic. There was no trace of œdema anywhere. His temperature, pulse, and respiration rates were natural. The lungs were natural; the heart was considerably hypertrophied, but showed no signs of failing. The blood pressure was high. The urine averaged about 60 ounces per diem, and it contained 6 parts per thousand of albumen; no blood or pus, but many granular and hyaline tube casts were present. Albuminuric retinitis was found on ophthalmoscopic examination. The diagnosis was clearly chronic nephritis and uræmia. The condition did not improve under treatment; a fortnight later there was severe epistaxis, followed by hæmatemesis, the latter being no doubt due to blood that had been swallowed. The mental condition remained fairly good until near the end, except for the persistent headache and insomnia. Ten weeks from the first onset of the nausea and vomiting, which were the first symptoms of anything being wrong, he suddenly became comatose, had one uræmic convulsion, and was dead. There was not a trace of œdema even at the end.

At the autopsy the brain weighed 1,300 grains, and looked perfectly healthy; the cerebral vessels were not atheromatous. The lungs, liver, spleen, suprarenal glands, pancreas, stomach, intestines, and aorta were all natural. The heart weighed 16 ounces; the right side was perfectly normal, so also was the left auricle; all the valves were healthy, the coronary arteries were good; the only abnormal part of the heart was the left ventricle, which felt like a cricket ball, owing to its uniform hypertrophy; its wall was 1¼ inch thick, and there was no dilatation. The kidneys weighed 2⅓ ounces each; they were symmetrical small white organs. The capsules were thick and adherent, and when peeled off they left pale granular bumpy surfaces mottled with specks of red. On section the cortex of each kidney was found to be a mere rim, about 1 millimetre in width; the pyramids were also shrunken, but to a less degree, the prevalent colour being a dull white splotched with crimson. The arterioles were not thickened; the pelves of the kidneys were not dilated, but contained excess of fat. The ureters, bladder, urethra and testicles were all natural. The condition was obviously that of insidious chronic tubal nephritis.

The microscopical changes seen in sections of the kidneys were very similar to those in the first case. There was extreme interstitial fibrosis, with great irregularity of the tubules, and atrophy of many of them and of the glomeruli.

Case 3.—A man, aged 28, an ex-soldier who had served in India, but who had always remained strong and well, began to suffer from symptoms of "dyspepsia" two and a-half months before he died. He attended as an out-patient at a hospital, and was thoroughly examined; *the urine was quite free from albumen* and sugar; the heart seemed to be of normal size; he was a thin man, but rather pale; there was no œdema anywhere; the fundus oculi was natural, and the diagnosis of ordinary dyspepsia appeared to be correct. There seemed no reason even to suspect that the kidneys were wrong; fortunately the absence of albuminuria is not the rule, or else most of these cases would be missed. The epigastric pains, nausea, and vomiting increased in severity, but the patient kept up and about until a fortnight before he died. The vomiting was then so severe, and the man felt so weak, that he took to his bed. The urine was tested on several occasions; it was always abundant, pale, and of low specific gravity, but was never albuminous when examined. When he had been in bed with the gastric symptoms for twelve days he had a bright red hæmatemesis, the amount of blood not being great; this was the only hæmorrhage; next day he became comatose, with twitchings of the left arm, paralysis of the right arm, pin-point pupils, but no general convulsions. Twenty-four hours after he went into this uræmic coma he ceased to live. There was no œdema even at the end.

At the autopsy the brain was perfectly healthy; the heart was not enlarged; there was recent pleurisy over both lower lobes, a terminal affection; but all the other organs were natural except the kidneys. These were small and pale, similar to those of the two preceding cases, with adherent capsule, pale, irregular surface, narrow cortex, and lack of definition between the cortex and medulla. The rest of the genito-urinary organs were quite healthy. Microscopical examination of sections of the kidneys showed much interstitial fibrosis, atrophy of many of the tubules, dilatation of others, fatty degeneration of many of the epithelial cells that were still left, atrophy of some glomeruli, whilst others were almost healthy; there were hyaline casts within some of the tubules. Notwithstanding the absence of albuminuria, therefore, the condition was one of small white kidney of insidious onset in a young person who had never had any recognisable acute nephritis—that is to say, a case of "Rose-Bradford kidney"; the first symptoms being those of chronic uræmia, which later became acute, and soon ended in death.

